# Zinc Oxide/Molybdenum Disulfide as Nanocomposite for Multifunctional Sensor Prototype

**DOI:** 10.3390/mi16040358

**Published:** 2025-03-21

**Authors:** Netzahualcóyotl Palomera, Peter Feng

**Affiliations:** Department of Physics, University of Puerto Rico, San Juan, PR 00931, USA

**Keywords:** multifunctional composite structures, two-dimensional semiconductors, molybdenum disulfide, photodetection, gas chemical tracing

## Abstract

Different materials are studied for environmental gas sensors as well as photodetection prototypes. A ZnO/MoS_2_ p-n junction was synthetized to act as a multifunctional sensor prototype. After the ZnO was prepared on a silicon substrate by using DC sputtering at room temperature, molybdenum disulfide layers were spin-coated on a nanostructured zinc oxide flake-shaped surface to form an active layer. The heterostructure’s composite surface was examined using scanning electron microscopy, energy-dispersed X-ray, and Raman spectroscopy. Responses to light frequencies, light intensities, and gas chemical tracing were characterized, revealing an enhanced multifunctional performance of the prototype. Characterizations of light-induced photocurrents indicted that the obtained response strength (photocurrent/illumination light power) was up to 0.01 A/W, and the response time was less than 5 ms. In contrast, the gas-sensing measurements showed that its response strength (variation in resistance/original resistance) was up to 3.7% and the response time was down to 150 s when the prototype was exposed to ammonia gas, with the concentration down to 168 ppm. The fabricated prototype appears to have high stability and reproducibility, quick response and recovery times, as well as a high signal-to-noise ratio.

## 1. Introduction

For more than a decade now, there has been active research in multifunctional prototype fabrication based on composite materials. Multifunctional prototype developments in gas sensors and UV light detectors are incorporated in communications and air pollution detection [1,2,3,4,5,6]. Two-dimensional materials such as molybdenum disulfide and its composite heterostructures are being used in the development of multifunctional prototypes [7,8,9,10,11]. Composites metal oxides and semiconductors materials are one of the principal material components used in the development of multifunctional prototype technology [12,13,14,15]. Multifunctional composite materials and microstructures include application fields such as photosensors and structurally integrated electronic components [16,17,18,19,20].

Sensors based on composites materials with planar configurations have attracted a lot of interest, due to their high sensitivity in monolayer form, broad response range, vast selection of materials that can be formed into planar composites, and the formation of heterostructures [21,22]. Molybdenum disulfide is a very well-suited material for sensor fabrication due to its mechanical and electronic properties.

Various methods for MoS_2_ nanosheets synthesis have been reported in the literature, including the spin-coating technique, which can be used to achieve controllable and structural uniformity deposition [23,24,25]. Sensors based on two-dimensional MoS_2_ have been utilized for gas and light detection. Molybdenum disulfide with an oxide semiconductive heterostructure is suitable for ultra-broadband photocurrent detection.

Zinc oxide has recently been used as the material of choice for sensor research, because of its wide band gap, low cost, strong radiation hardness and high chemical stability. Its synthesis allows for the capability of tailoring the dimensional structure from nano- to micro polycrystallinity and is regarded as a very promising candidate for UV photodetectors [26,27,28,29,30,31,32,33,34,35,36]. Prototypes with zinc oxide/molybdenum disulfide composite structures have shown photoelectric conversion efficiency, a photovoltaic mode, and hole transportation properties [37,38,39]. The ZnO sensor also shows a decent response even at room temperature, and metal oxide thin films have proven advantageous for electronic prototypes such as gas sensors [40,41,42,43].

Our present work involved gas chemical tracing and measurements of photo-response capability. The characterization of the composite revealed an improved heterostructure consisting of randomly orientated rough and irregular clusters combined with microstructured flakes of metal oxide components. This provides an electronical interface structure, allowing for the production of a high-performance multifunctional sensor prototype. The features achieved include multifunctionality: the chemical tracing of ammonia, and capability for UVA and deep UV photo sensing. An additional photovoltaic response was also ensured as a proof of concept for a transistor element prototype in the complete wide visible-light spectrum.

## 2. Materials and Methods

### 2.1. Synthesis

The microstructure zinc oxide (ZnO) surface was deposited on 2 cm by 1.5 cm silicon (Si) substrates, using direct-current (DC) sputtering at room temperature (100 Watts under an argon atmosphere of 1.7 × 10^−2^ Torr), using a ZnO sputtering target (from Plasmaterials, Livermore, CA, USA). The deposition was carried out for two consecutive lapses of two minutes, with the target and the substrate being 3.5 cm apart.

Molybdenum disulfide (MoS_2_) layers were deposited on the Si/ZnO substrate using a simple spin-coating technique, with a commercial atomically thin layer MoS_2_ dispersion with LiOH as solvent (concentration of 1 mg/mL from XF135, XFNANO Materials Co. Ltd., Nanjing, China). Prior to the deposition of film, the MoS_2_ solution was ultrasonicated at 200 W for one hour at 60 Hz amplitude in 10 s intermittent pulses, to break agglomerates and have distribution uniformity. Each spin-coating run was for 20 s with a spin rate of 3000 min^−1^. Ten coatings were used to increase the number of two-dimensional MoS_2_ sheets on the surface of the Si/ZnO substrate. A soft baking step of 3 min at 120 °C was applied between each layer-deposition step.

### 2.2. Set-Up

**Figure 1:**
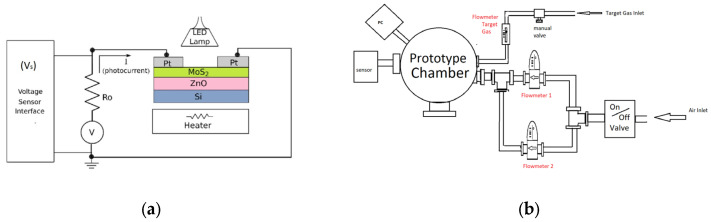
(**a**) Circuit diagram for Zn/MoS_2_ composite implementation as photodetector prototype; (**b**) set-up for gas chemical-tracing characterization.

After having completed the synthesis of the Si/ZnO/MoS_2_ active layer, the sample was exposed to DC sputtering (platinum sputtering target from Plasmaterials) to deposit two electrode nodes. The series circuit connected to an external resistivity of 1.7 KΩ was incorporated as a photo-sensing prototype, which can be seen in Figure 1a. The set-up for the prototype as a small-volume pollution sensor system can be seen in Figure 1b. Two flowmeters were used for testing the target pollution gas, an air mixture, with an air flow of 74.16 L per minute (LPM) in each one, giving a total of 148.32 LPM. For the target gas, the flowmeter was set at a range between 0.025 and 0.5 LPM.

## 3. Results

### 3.1. Structure Characterizations

**Figure 2:**
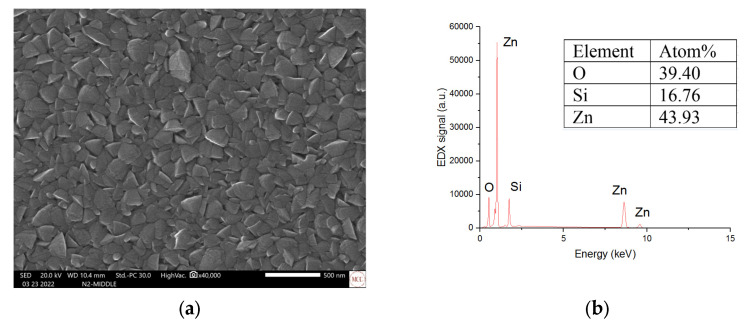
(**a**) SEM images of microstructured ZnO and (**b**) EDX of microstructured ZnO.

**Figure 3:**
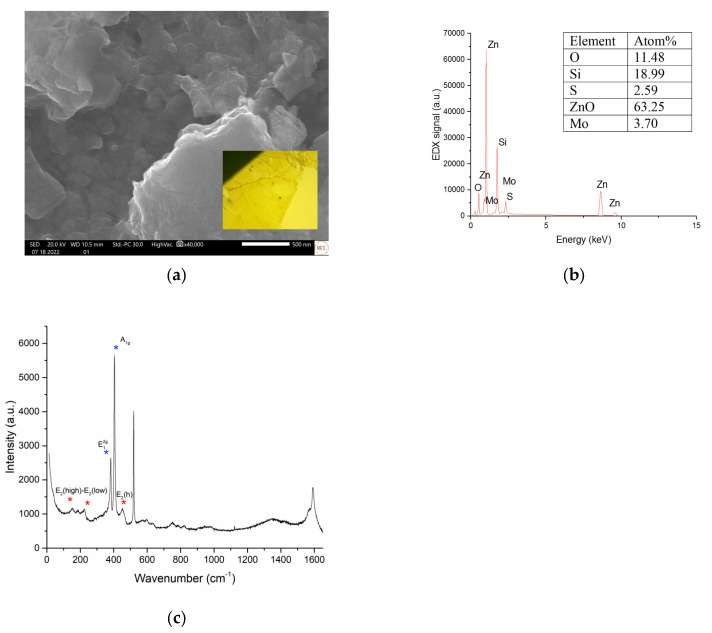
(**a**) SEM images of ZnO/MoS_2_ heterojunction; (**b**) EDX of ZnO/MoS_2_ heterojunction; (**c**) Raman spectra of ZnO/MoS_2_ sample.

Figure 2a shows a scanning electron microscope (SEM) image of the sputtered ZnO nanostructured flakes, with an average length of 200 nm prior to MoS_2_ deposition. Atomic percentages of the nanostructured ZnO surface are shown in Figure 2b, as 43.93 and 39.40, respectively, with an Zn/O ratio of 1.13. The fabricated Si/ZnO substrate was then used with a spin-coated MoS_2_ solution to form layers of randomly orientated clusters. The use of a relatively physicochemical method such as spin coating is due to the weak interlayer interaction of the materials. As shown in Figure 3a, SEM structural characterization confirms the presence of few-layer nanosheets [44]. An optical microscope image (5 μm × 5 μm) of a two-dimensional MoS_2_ sample used in the process is included (inset). The nanosheets have a thickness of around 0.65 nm for the atomic layer, with clusters between 800 nm and 200 nm [45]. After heterostructure formation, the atomic percentages with relative MoS_2_ signal are 0.67 and 1.31, with an S/Mo ratio of 1.95, as shown in Figure 3b. The presence of S and Mo elements indicates that the successful growth of the MoS_2_ and element ratios between the O/Zn atoms remained almost unchanged in the heterostructure spectra. Figure 3c shows Raman spectrum measurements performed on the sample with a triple monochromator and a 514 nm Ar+ ion laser beam. Raman activates E_2g_^1^ at 379.1 cm^−1^ and A_1g_ at 400.5 cm^−1^ appears, indicating the MoS_2_ peak. The active narrow Raman E_2g_ (high) peak has a well-defined ratio. This is in good agreement with the data obtained using EDX on a quantitative analysis of chemical composition, where the ratio remains unchanged. The wavenumber difference of 20 cm^−1^ between the E_2g_^1^ and the A_1g_ peaks suggested that the average sheet thickness of each sample was only two atomic layers [46]. E_2_ (high)-E_2_ (low) ZnO Raman scattering spectral lines were also observed.

### 3.2. Visible-Light Detection

#### 3.2.1. Wavelength Response

**Figure 4:**
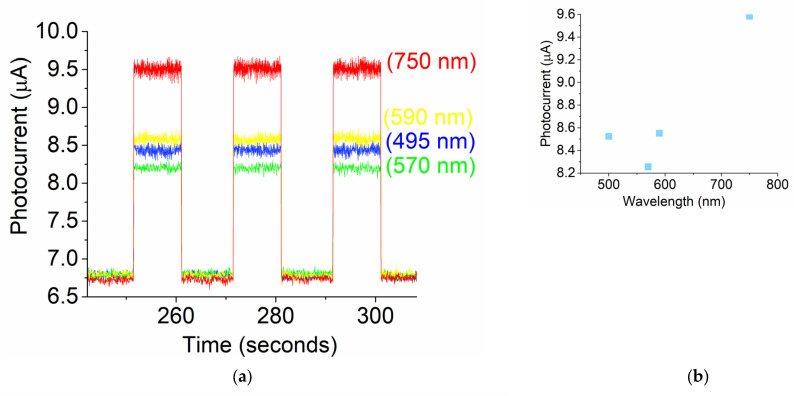

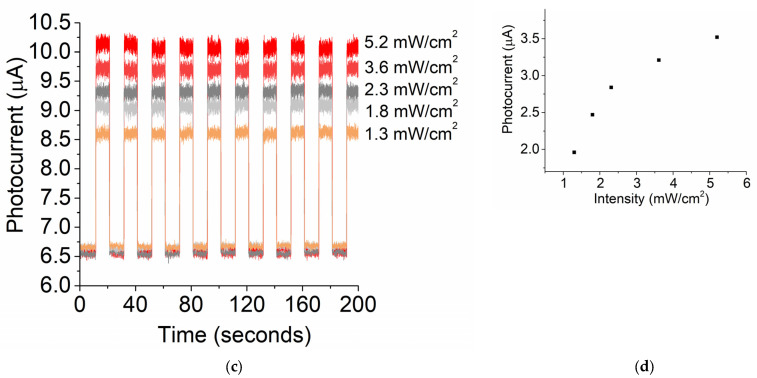
(**a**) Different broadband light-induced photocurrents during a cycling test at an intensity of 5.2 mW/cm^2^; (**b**) photocurrent vs. wavelength; (**c**) red light-induced photocurrents during a cycling test at different illumination intensities; (**d**) photocurrent vs. red light intensity.

A single layer of two-dimensional molybdenum composites for field-effect transistors has attained a ~400–700 nm range [47]. The LED light source used for this test is a single collimated beam source with different λ between 450 nm and 750 nm. Figure 4a shows different light-induced photocurrents on the prototype, operating at zero bias and room temperature. The light illumination intensity was 5.2 mW/cm^2^. The signal-to-noise ratios for blue, green, yellow, and red were 1.54, 1.44, 1.61, and 2.02, respectively. As indicated by the data, the prototype provided a highly stable baseline and an easily repeatable signal, with the strongest photo response at the red wavelength of 750 nm (Figure 4b). The response time of the photocurrent is almost immediate once the light is turned on or off.

#### 3.2.2. Intensity Effect on Photocurrent

Figure 4c shows effect of light intensity on light-induced photocurrent of prototype exposed to a red LED light during cyclic tests with a 10 s period at room temperature and zero bias. The illumination intensity of the sensor increased inversely square (1/r^2^) as the distance between the prototype and the light source was diminished. By varying the distance between the prototype and the LED light source, illumination intensity on the surface of active layer was controlled. The generated photocurrent directly associated with light absorption showed a fast response as the light source was turned on and off. An increase in illumination intensity from 1.3 to 5.2 mW/cm^2^ resulted in an increase in light-induced photocurrent from 1.96 to 3.52 µA, as shown in Figure 4d.

#### 3.2.3. Bias Effect

**Figure 5:**
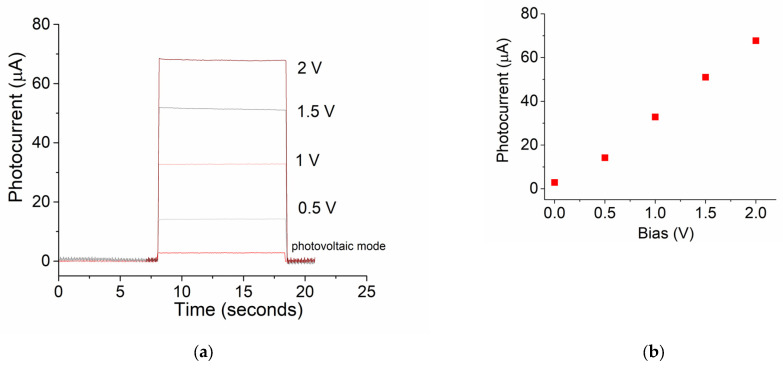

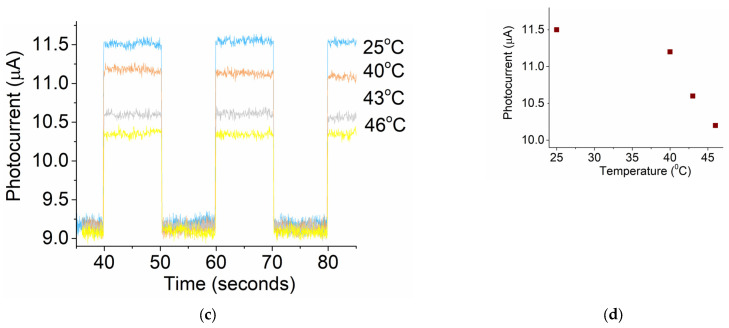
(**a**) Photocurrent time curves for red light with bias from photovoltaic mode to 2 V; (**b**) photocurrent vs. bias; (**c**) photocurrent time curves for red light at temperatures from 25 °C to 46 °C; (**d**) photocurrent vs. temperature.

The effect of bias on the induced photocurrent was studied under red-light illumination at an intensity of 5.2 mW/cm^2^. Figure 5a shows generated photocurrents at bias of 2 V, 1.5 V, 0.5 V, and the photovoltaic mode. The transient dynamic behavior remained similar no matter the change in bias, and there was a proportional relationship between the changes in biases. As seen in Figure 5b, the induced photocurrent increases linearly with an increase in the bias voltage.

#### 3.2.4. Temperature Effect

Temperature-correlated measurements were performed in a range between 25 °C and 46 °C, to study the photocurrent behavior. The prototype revealed excellent stability in the chosen temperature range. This can be verified given that the photovoltaic baseline showed an increase in value, while the obtained current to light remained constant in each chosen temperature as show in Figure 5c. A high operating temperature normally would affect light absorption, resulting a decrease in the photocurrent, as shown in Figure 5d. The signal-to-noise ratios of 25 °C, 40 °C, 43 °C, and 46° were 1.92, 1.65, 1.39 and 1.36, respectively, showing a decrease in spectral sensitivity with increasing temperature.

#### 3.2.5. Time Response

**Figure 6:**
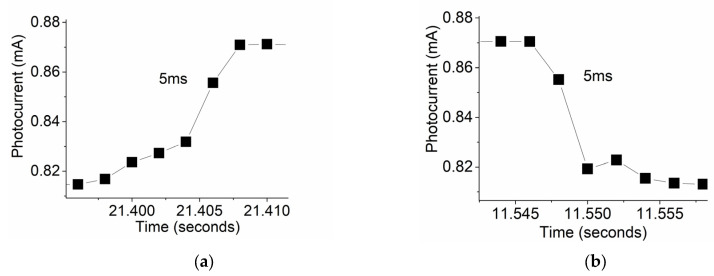
(**a**) Response and (**b**) recovery times of a ZnO/MoS_2_ prototypic photodetector at a 2 V bias.

Characterizations of response time of the prototype exposed to visible red light was also performed at a bias of 2 V, with an excellent performance in the generated photocurrent and response time. The data obtained in Figure 6a,b show a response and recovery time of 5 milliseconds.

### 3.3. Ultraviolet Photodetection

#### 3.3.1. Deep UV Photoelectric Properties

**Figure 7:**
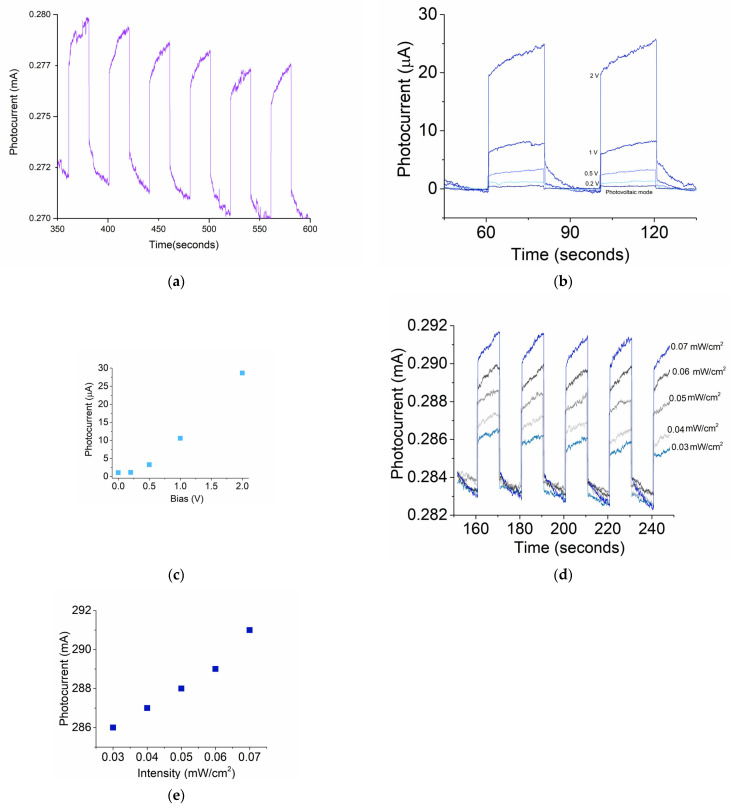
Time evolutions of (**a**) 0.07 mW/cm^2^ deep-UV-induced photocurrent; (**b**) UVA-induced photocurrents at different biases; (**c**) photocurrent vs. bias; (**d**) time evolution of UVA-induced photocurrents at different illumination intensities; and (**e**) photocurrent vs. UVA intensity.

UV radiation in the solar-blind region between 220 nm and 280 nm wavelengths is called deep UV. Deep UV detection at λ = 264 nm was performed in a circuit scheme, as shown in Figure 1a. The transient dynamic behavior is shown in Figure 7a, with a bias of 1 V. Photocurrent signals display a signal-to-noise ratio of 1.046095.

#### 3.3.2. UVA Bias Effect

The UV spectrum is generally divided into three bands: A, B, and C. The band at 320 nm is in the UVA region. UVA photoelectric characterizations of the prototype’s properties were performed at a wavelength of 320 nm. The effect of bias on the generated UVA photocurrent was studied at an intensity of 0.07 mW/cm^2^. Figure 7b shows the generated photocurrents at biases of 2 V, 1 V, 0.5 V, 0.2 V and photovoltaic mode. The response and recovery times remained similar no matter the change in bias. A slightly nonlinear relationship between the bias and the ultraviolet-induced photocurrent was observed as shown in Figure 7c, indicating a different bias effect on the response of the prototype exposed to visible- and UV light illuminations.

#### 3.3.3. UVA Intensity

The characterizations of ultraviolet-light-induced photocurrent indicated an obtained response strength (photocurrent/illumination light power up to 0.01 A/W, for UVA at a bias of 1 V). The intensity effect on the responses of the prototype exposed to UVA can be seen in Figure 7d for intensity illumination between 0.03 and 0.07 mW/cm^2^. The photocurrent generated in function of illumination intensity can be seen in Figure 7e.

### 3.4. Chemical-Tracing Gas Detection

Besides photoresponsivity, catalytic activity on the surface of the composite heterojunction was investigated to prove the multifunctionality of the prototype. For this, the composite was implemented into a gas-sensing prototype. The performance of the circuit is measured as gas response (GR), and is defined based on the variation in chemiresistivity. It is presented in Equation (1):

GR% = (ΔR/R_0_)% = (R_g_ − R_0_/R_0_)%, (1)

where R_g_ is the resistance of ZnO/MoS_2_ in the target gas environment and R_0_ as the initial resistance without target gas.

Preliminary experiments were carried out at a low concentration of ammonia as the target gas. During gas-sensing experiments, anhydrous ammonia was selected and its gas flow rate into the chamber was set to be 0.025 LPM. Regular air was used as a mixture to dilute the original ammonia gas. The air flow rate was set to be 148.32 LPM. Correspondingly, the obtained concentration (N) inside the chamber was 168 parts per million (ppm), as evaluated by Equation (2):

N = (0.025/(148.32 + 0.025) × (1 × 10^6^) = 168 ppm. (2)

**Figure 8:**
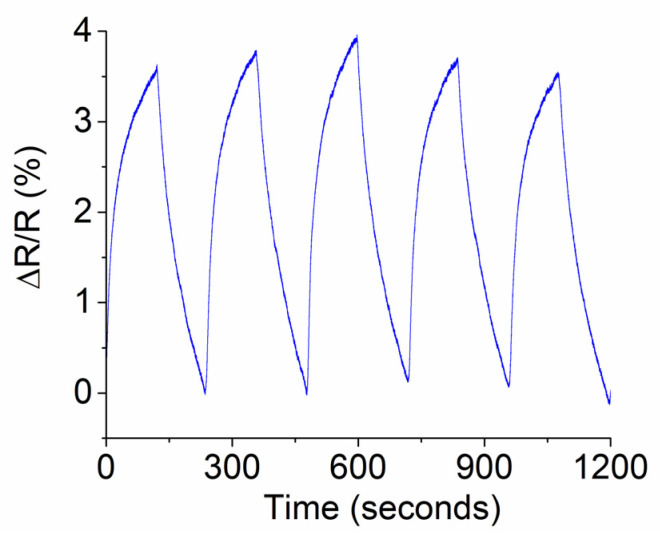
Chemical-tracing transient response behavior of prototype exposed to 168 ppm ammonia as target gas.

As seen in Figure 8, an ammonia gas response of 3.7% has a rise and recovery time of 150 s. The response time for the gas sensor as compared to that of the light-induced photocurrent was long, suggesting that the process of adsorption and desorption of gas molecules on the surface of the active layer was slow.

Table 1 compares the performance of active layers and the ammonia-sensing devices in this work.

The interaction of ammonia with ZnO contribution [49] is presented in Equation (3):

4NH_3_ + 3O_2_^−^ = 2N_2_ + 6H_2_O + 6e^−^. (3)

The contribution of MoS_2_ with ammonia [50] is presented in Equation (4):

2NH_3_ + 3O^−^ = N_2_ + 3H_2_O + 3e^−^. (4)

## 4. Discussion

We have successfully incorporated a ZnO/MoS_2_ active layer onto a silicon wafer surface using direct-current plasma sputtering and a spin-coating method. Raman, EDX, and SEM reveal the formation of nanostructured metal oxide as well as a composite heterojunction. The ZnO/MoS_2_-based prototype has been demonstrated for its multifunctional sensing applications. The fabricated prototype showed an excellent signal-to-noise ratio and excellent properties, including multifunctionality, low-cost fabrication, fast photo-response time, highly stable baseline, good repeatability, photovoltaic behavior, broadband spectra photodetection, and pollution gas chemical-tracing detection features. Different biases, illumination intensities, light frequencies, operating temperatures, and ammonia gas detection methods were studied. An enhanced performance of the prototype was obtained even though the methods used in preparations of multifunctional nanomaterials were simple and cost-effective. Work is in progress to further optimize the structural parameters and morphology of further material into composites and multifunctional sensing applications.

## Figures and Tables

**Table 1:** Performance in terms of ammonia gas sensing of other active layers.

Active Layer	Fabrication Method	ΔR/R_0_	Concentration
ZnO [48]	Spin Coating	38%	400 ppm
ZnO/MoS_2_ [This Work]	Sputtering + Spin Coating	3.7%	168 ppm
MoS_2_ [49]	Electrospinning	40%	200 ppm
ZnO/MoS_2_ [50]	Chemical Vapor Deposition	18%	100 ppm

## Data Availability

Dataset available from the authors on request.
